# Healthcare experiences and the cycle of genomic healthcare disparities: A cross-sectional study utilizing the ‘*All of Us*’ research program

**DOI:** 10.1007/s12687-026-00921-8

**Published:** 2026-07-22

**Authors:** Megan D. Johnson, Aubrey Hite, Jennifer Richmond, Emily C. Lisi, Hannah C. Ainsworth

**Affiliations:** 1 Genetic Counseling Graduate Program, Wake Forest University School of Medicine, Winston-Salem, NC USA; 2 Atrium Health Levine Children’s Hospital, Charlotte, NC USA; 3 Department of Pediatrics, Section on Medical Genetics, Atrium Health Wake Forest Baptist, Winston-Salem, NC USA; 4 Department of Neurology, Atrium Health Wake Forest Baptist, Winston-Salem, NC USA; 5 Department of Social Sciences and Health Policy, Division of Public Health Sciences, Wake Forest University School of Medicine, Winston-Salem, NC USA; 6 Department of Biostatistics and Data Science, Division of Public Health Sciences, Wake Forest University School of Medicine, Winston-Salem, NC USA; 7 Center for Precision Medicine, Wake Forest University School of Medicine, Winston-Salem, NC USA

## Abstract

Our understanding and utilization of genetics in healthcare continues to increase, but historically excluded and underrepresented groups (e.g., racial minority groups, those living in rural areas, and individuals from lower socioeconomic backgrounds) are often left behind due to barriers in accessing genetic healthcare and research. Although research priorities have shifted toward greater inclusion, underrepresentation persists, potentially driven by ongoing negative healthcare experiences that compound barriers to accessing genetic research and healthcare services, creating a cycle of genomic healthcare disparities. However, there is limited research about how healthcare experiences may contribute to genomic healthcare disparities. We examined this knowledge gap by using data from the All of Us Research Program to investigate healthcare experiences of participants from underrepresented groups as well as participants with clinically diagnosed genetic conditions. We explored three survey questions which documented self-reported experiences when receiving healthcare (i.e., inclusion in medical decisions, being treated with respect by providers, and receiving easy to understand medical information). Survey question responses were analyzed within a regression framework which included self-identified race, age, gender identity, health insurance status, education and income levels, having a diagnosed genetic condition, and rural location as predictors. Results suggest many populations (e.g., minoritized racial and gender groups, individuals who have had to delay care due to living in a rural area, and those with genetic conditions) face inequities in healthcare experiences. These findings support the proposed conceptual model of a genomic healthcare disparity cycle and the need to address these negative healthcare experiences to break the cycle.

**Supplementary Information:**

The online version contains supplementary material available at https://doi.org/10.1007/s12687-026-00921-8.

## Introduction

The role of genetics in clinical medicine has drastically increased over the past few decades, a trend which is expected to continue as we advance towards precision medicine (Zeggini et al. 2019). Genetics research has historically centered around individuals of European descent without consideration of, or attention to, barriers to research participation faced by patients from underrepresented groups, including the long-standing harm and distrust caused by unethical research practices (Washington 2008; Dolan and Bell 2024). Many ancestral and ethnic groups continue to be underrepresented in genetics research leading to recent medical advances that are not well-translated across global populations, such as polygenic risk scores (Manrai et al. 2016; Martin et al. 2019). Of note, it is important to distinguish ancestry, or the passing down of genes through generations, from race, which is a sociopolitical construct to classify individuals based on beliefs about their shared ancestry (Committee on the Use of Race, Ethnicity, and Ancestry as Population Descriptors in Genomics Research et al., 2023; Supplementary Table 1). Although race must be defined to identify whether there are racial disparities in research, race cannot be utilized to infer genetic makeup. Conversely, ancestry is often used to infer genetic similarity from geographical origins (Yudell et al. 2016). Beyond racial inequities, inequities in research participation have also been found among individuals with lower socioeconomic status, living in rural areas, and with less educational attainment (Lemke et al. 2022). As precision medicine continues advancing towards developing effective biomarkers to facilitate medical diagnoses and interventions, research has shown limited and even harmful applications of these advances towards historically underrepresented groups (Manrai et al. 2016; Martin et al. 2019; Medford and Moy 2024). For example, population-level use of genetic testing procedures that were developed and validated primarily among individuals of European-descent may lead to missed or incorrect diagnoses among other populations (Florentine et al. 2022; Manrai et al. 2016). Accordingly, genetics researchers have taken steps globally to increase equitable participation in research (Fatumo et al. 2022; Lemke et al. 2022), but improving equity in healthcare goes beyond representation in research (Khoury et al. 2022).

Individuals from underrepresented groups face barriers at every step in genomic healthcare. A recent literature review by Fraiman and Wojcik (2021) found that providers (e.g. primary care physicians, specialists) were less likely to refer patients identifying as racial/ethnic minorities to genetic specialists (e.g. medical geneticists), and that genetic specialists may be less likely to suspect a genetic syndrome and order genetic testing in these groups. Although not explicitly explored in our study, it has also been suggested that families with limited English proficiency may not be referred to genetic specialists unless the patient has more severe features than seen in referrals for English-speaking families (Wojcik et al. 2023). Further, most genetics providers primarily practice in large academic medical centers in major cities, which can decrease access to care for those living in rural areas due to the necessity of transportation often across a long distance (Lyon et al. 2025). Even if underrepresented individuals overcome each of these described barriers and complete genetic testing, variants found during testing are more likely to be difficult for labs to interpret due to the lack of diversity in genomic reference datasets (Long et al. 2022). Genetic counselors play a unique role in increasing access to genetic testing due in part to the abundance of genetic counselors in clinical practice compared to the number of medical geneticists; there were approximately 6,641 genetic counselors and 2,316 medical geneticists in the U.S. in 2023, both increasing from 2019 (Cosgrove et al. 2020; Lyon et al. 2025). Even as the genetic counseling profession continues to grow, they are vastly outnumbered by the individuals who would benefit from genetic healthcare. Furthermore, the genetic counseling profession is pushing for Medicare recognition and the ability to be reimbursed for services. Although this change could incentivize institutions to hire more genetic counselors to provide care to these individuals, financial barriers for uninsured or underinsured patients will remain.

Although previous research has identified several barriers to genomic healthcare and research disparities, there is limited research about how prior experiences receiving and accessing healthcare may contribute to these disparities (Nguyen et al. 2020). Indeed, when patients encounter disrespect or exclusion from decision-making during medical appointments, avoiding future healthcare encounters and declining research participation can become a rational response to protect themselves from additional negative experiences (Richmond et al. 2024). Furthermore, such negative healthcare experiences can foster medical distrust, which is a large factor contributing to underrepresentation in genetics research (Angelo et al. 2022). While sometimes utilized interchangeably, we emphasize the distinction between medical mistrust and medical distrust. Medical mistrust typically refers to an overall sense of suspicion, unease, and broad intuition that medical providers or institutions in general will provide care that does not meet a patient’s expectations or may actively cause harm. Medical distrust refers to a suspicion or unease about specific healthcare providers or organizations, often in response to concrete prior negative experiences (Griffith et al. 2021; Richmond et al. 2024). Collectively, patients in underrepresented groups disproportionately encounter negative healthcare experiences with specific providers and/or organizations, potentially exacerbating medical distrust and genomic inequities (Richmond et al. 2024). To improve genetic health equity, it is critical to understand the factors contributing to perpetual disparities, such as patient healthcare experiences. We therefore utilized The All of Us research program (The All of Us Research Program Investigators 2019) to explore the larger picture of recent healthcare experiences for participants from underrepresented groups as well as participants with clinically-diagnosed genetic conditions. We also conceptually explored how healthcare experiences operate within and may contribute to a larger cycle of genomic healthcare disparities.

## Methods

### Conceptual framework

**Fig. 1:**
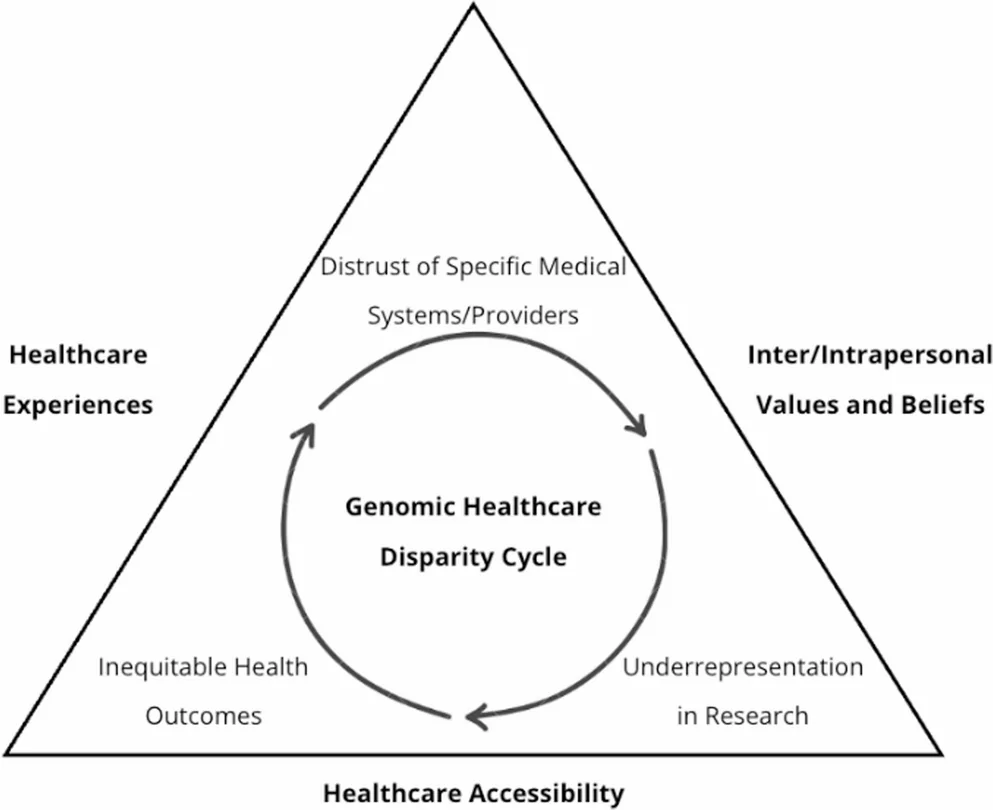
Conceptual Model of the Genomic Healthcare Disparity Cycle. Diagram depicts an inner cycle illustrating how distrust, underrepresentation in research, and inequitable health outcomes mutually reinforce one another and perpetuate the Genomic Healthcare Disparity Cycle. The outer triangle represents the structural and contextual factors (i.e., inter/intrapersonal values and beliefs, healthcare accessibility, and healthcare experiences) that both shape and are shaped by this cycle. Collectively, the diagram reflects the complexity and multidirectional nature of genomic healthcare disparities.

We adapted a conceptual framework based on the model of medical distrust proposed by Angelo et al. (2022) and a review of current literature to guide the focus of this study (Fig. 1). The model of medical distrust proposes that patient characteristics, including sociodemographic characteristics and healthcare accessibility, influence medical distrust and therefore the experience and outcome of clinical encounters. Our novel framework depicts a proposed cycle for the ongoing presence of genomic healthcare disparities, incorporating the role of medical distrust as well as the inequity in accessibility of genetics and informative genetic testing results.

For an example of the proposed conceptual model, individuals who distrust the medical system or their providers are historically less likely to participate in research due to the barriers involved, which in the context of genetics has been shown to result in increased variants of uncertain significance (VUS) for these groups due to limited data to assess pathogenicity such as population frequency (Chapman-Davis et al. 2021). Medical management decisions should not be based on a VUS finding alone, but instead falls on providers. While there are guidelines in specialties such as oncology where providers can base screening and medical management on family or personal history for individuals with VUS results, other specialties such as cardiology do not have clear guidelines (Makhnoon et al. 2021; Muller et al. 2020). Therefore, care may vary greatly by provider and even for each patient under their care, leading to wide-ranging experiences for patients. Providers may feel unsure about the best next steps to navigate uncertain genetic risk while also avoiding unnecessary and excessive management, but their recommendations can directly impact how patients respond to their VUS result (Macklin et al. 2019; Mighton et al. 2021). This uncertainty may also elicit provider biases (e.g., about patient ability to understand or follow medical recommendations) that may disproportionately impact patients from underrepresented groups. As a result, regardless of an eventual genetic reclassification to (likely) benign or (likely) pathogenic, the increased likelihood of a VUS result can directly impact an individual’s healthcare experience and trust in the medical system as each person’s care is managed differently, further perpetuating this cycle and disproportionately impacting underrepresented groups. It is also critical to consider the factors external to this process, such as interpersonal and intrapersonal beliefs that play a large role in how individuals view the medical system and participation in research (Pratt et al. 2020; Shepherd et al. 2018). Overall healthcare accessibility also plays a role in the ability of an individual to participate in research as well as improving their personal health outcomes. For instance, excessive burden on patients to access health providers may lead to decreased seeking of necessary care and inability to access the academic medical centers where research studies are often based.

We leveraged this conceptual model to guide our study by informing variable selection and analytical approach, specifically directing our focus to sociodemographic characteristics that may influence healthcare experiences within genetic care contexts. This focus was motivated to help address a current knowledge gap in literature and complement existing research that has focused on the impact of healthcare accessibility in both research participation and health outcomes. The model highlights that healthcare experiences are both an outcome of and contributor towards health inequities, recognizing that healthcare experiences occur within a larger system. We further apply this framework in our discussion to interpret the study results, describe how healthcare experiences contribute to inequitable outcomes, assess implications for genetic healthcare delivery, and identify areas for future research (Fig. 1).

### Data source

This secondary data analysis was performed utilizing the All of Us Database v7 (enrollment cut off July 1st, 2022; released September 10th, 2024), which is an ongoing National Institutes of Health-sponsored resource developed with the purpose of building a dataset representative of the United States population to inform studies on various health conditions (The All of Us Research Program Investigators 2019). To accomplish this goal, All of Us works to address several of the previously discussed barriers and concerns of underrepresented groups through building connections with communities and healthcare systems, establishing a high standard of data security to ensure privacy, centering research participants as partners, and increasing transparency in the research process. This has included making materials available in both English and Spanish to increase access, including the survey analyzed in this study. Additionally, *All of Us* is transitioning from returning only research results to also providing clinical genetic testing results to study participants including information about treatable or preventable health conditions and medication metabolism. As a result of these efforts, the *All of Us* database is more inclusive of underrepresented groups, enabling a unique opportunity to assess diverse participant experiences when seeking healthcare. Many of the participants in the *All of Us* Program were informed about the study by providers at large academic medical centers, VA medical centers, and community health centers throughout the United States, but participants could also join directly by signing up through their website.

In the current study, we analyzed secondary data from *All of Us* participants who completed the “Healthcare Access and Utilization” (HCAU) survey, which is publicly available on *the All of Us* research hub (https://researchallofus.org/). This is an optional survey that participants can fill out after joining the *All of Us* research study, participants who did not complete this survey was the only initial exclusion criteria for analyses. Other reasons for exclusion are described below.

### Outcome variable

The primary outcome of this study was self-reported healthcare experiences. Given the unique role that genetic counselors play in expanding access to genetic healthcare and patient-oriented care, three survey questions were selected based on how strongly they correspond to several of the practice-based competencies for genetic counselors: facilitating decision-making and ensuring informed consent for testing, building a working alliance with patients, and tailoring communication and resources to aid in making the complexities of genetics easier to understand (Accreditation Council for Genetic Counseling, 2023). These three survey questions focused on specific areas of the experience participants have when they access healthcare: (1) “How often did your doctors or health care providers ask for your opinions or beliefs about your medical care or treatment? For example, what kind of tests, procedures, or medications you prefer.”, (2) “How often were you treated with respect by your doctors or health care providers?”, and (3) “How often did your doctors or health care providers tell or give you information about your health and health care that was easy to understand?” These three survey questions shared answer options which were: “Always”, “Most of the Time”, “Some of the Time”, and “None of the Time”.

### Predictor variables

Predictor variables were selected based on prior literature in the realm of genetic inequity and our conceptual model of the Genomic Healthcare Disparity Cycle. Presence of a diagnosed genetic condition in the electronic health record (as identified by *All of Us* through the enrollment process) was included as a variable in our analysis to facilitate a comparison of experiences between individuals who have and have not been clinically diagnosed with a genetic condition. We note, this category does not include participants who may receive a new genetic diagnosis through the *All of Us* study; genetic diagnosis (based on *All of Us* genetic data results) was not available as of the v7 data release.

Sociodemographic predictor variables included the participant’s self-identified race, age, gender identity as well as income and education level. Of note, options for participants when identifying race included: “American Indian or Alaska Native”, “Asian”, “Black, African American, or African”, “Middle Eastern or North African”, “Native Hawaiian or other Pacific Islander”, “White”, and “None of these fully describe me”, with the option to select more than one. The following definitions were created and implemented by *All of Us*: (1) Participants who selected groups other than White, Asian, Black or African American were condensed as “Another single population.” (2) Participants who selected more than one group are considered “More than one population,” and individuals who selected None of these fully describe me were grouped as “None of these.” (3) Participants could describe their gender identity as: “man”, “woman”, “non-binary”, “transgender”, “none of these describe me”, and “prefer not to answer”. However, these identities were collapsed by *All of Us* into three categories: “male”, “female”, and a third group of the remaining answers labeled “not man only, not woman only, prefer not to answer, or skipped.” *All of Us* collapses data for groups where there are fewer respondents selecting those answers to protect participant privacy. Due to this survey limitation, participants who identify as non-binary, transgender, none of the other options, or who elected not to share their gender identity are represented in this study as “Other Identity or Prefer Not to Answer.”

Predictor variables were coded as follows. Self-identified race and gender identity were retained as nominal, multi-level, categorical variables. For statistical modeling of these two variables, white and female were selected as references (i.e., odds ratio presented as OR = 1 in results) due to the increased representation of these groups in genetic studies; this enables the statistical model to capture the impact of other groups, which are historically less represented. Age was ordinally coded from 1 to 3 (18–44 years, 45–64 years, and 65+), with higher scores corresponding to a higher age. Genetic condition was coded as no = 0 and yes = 1. Education level was ordinally coded from 1 to 4 (less than a high school degree or equivalent, 12th or GED, 1–3 years of college after high school, and college graduate or advanced degree), with the higher scores corresponding to increased education levels. Finally, annual income level was also ordinally coded from 1 to 3 (up to 35k, 35k-100k, and over 100k), with higher scores representing higher income levels.

In consideration of primary access and participation barriers identified in previous research, we also included health insurance coverage and whether the participant identified as being from a rural area as predictors (Dean et al. 2017; Goldenberg et al. 2013). While the HCAU survey includes two questions about health insurance, these are more focused to specific scenarios (e.g., whether a health care office did not accept coverage; and if coverage is better, worse, or the same compared to the previous year). For this reason, participant health insurance coverage was collected from the demographics survey with 0 = no and yes = 1. Whether an individual lived rurally was not directly represented by any *All of Us* research questions, so we used the following HCAU survey question as a proxy: “There are many reasons people delay getting medical care. Have you delayed getting care for any of the following reasons in the PAST 12 MONTHS? You live in a rural area where distance to the healthcare provider is too far.” Rural location was encoded as a binary variable 1 = yes (delayed care) and all other responses (no, “did not know”, or “skipped”) were encoded as 0. All variable encodings are summarized in Supplementary Table 2.

### Statistical analysis

Initial summary statistics revealed that survey questions 2 and 3, assessing patient experiences being treated with respect and receiving easy to understand healthcare information, exhibited disproportionate counts across the four answer possibilities and were not appropriate for analysis as an ordinal outcome using a cumulative logit model. This necessitated collapse of responses into a binary categorization. Survey question 1, which featured greater counts across all four possible responses, was analyzed as both an ordinal outcome using a cumulative logit model and a binary outcome using logistic regression. For binary outcomes, survey responses “Always” and “Most of the Time” were encoded as 1 and “Some of the Time” and “None of the Time” were encoded as 0. Primary inference is based on these binary outcomes using logistic regression models which were computed separately for each of the three survey questions. Odds ratios and 95% confidence intervals are reported, and overall model performance was assessed via the area under the Receiver Operating Characteristic curve (c-statistic). Statistical significance was assigned as *p* < 0.002, using a Bonferroni adjustment considering three models and eight predictors. For comparison with the multivariable models, simple logistic regression models were also computed for each of the predictors and outcome questions. For cumulative logit models, the proportional odds assumption was checked for each predictor variable.

Apart from “rural location”, all analyses were filtered to ensure complete data; participant answers of “Skip” or “Don’t Know” were considered missing data and excluded. Our goal was to evaluate effects among participants with self-perceived impact of their rural locality on healthcare. Thus, as previously described, for rural location, “Skip” and “Don’t Know” were combined with participants who answered ‘no’ and coded as 0. To evaluate potential bias in this approach, models were reanalyzed with rural location as a three-level nominal variable but the overall inference within rural location and across other predictors remained unchanged. Thus, the binary analysis is reported. Pairwise concordance of responses was also assessed using Cramér’s V, as derived by the Pearson chi-square. Percentages of concordant (both responses 0-negative experience or both responses 1-positive experience) and discordant answers were also summarized. All data quality control, analysis, and visualization were performed within the *All of Us* researcher workbench using R Studio and SAS Studio.

## Results

Of the 334,156 participants in the *All of Us* v7 data release, 165,812 had completed the “Healthcare Access and Utilization” (HCAU) survey (Table 1), which presented some differences in demographics between the cohort used for analyses and the *All of Us* v7 data (Supplementary Table 3). We describe the overall HCAU cohort demographics using percentages and whether this presents an increase (+) or decrease (-), relative to the *All of Us* v7 data. The overall cohort predominantly identified as female (63.7%; +4.1%), had attained at least some college education (87.4%; +12.3%), and had health insurance coverage (96.4%; +3.9%) (Table 2). Most participants did not have a genetic condition (95.7%; -0.4%) and did not report delay in care due to living in a rural area (93.2%; 0% —only reported in HCAU). While there was a wider range for ages and income levels, the largest groups were age 65+ (37.8%; +4.7%) and an annual income over 100k (34.1%; +9.3%). Self-identified race highlighted that most participants identified as White (81.0%; +13.2%), with the next most represented group being Black or African American (11.1%; -12.3%).

**Table 1:** Response summaries for selected questions of the Healthcare Access and Utilization survey

Question	No Response	None or Some of the Time	Most or All of the Time
How often did your doctors or health care providers ask for your opinions or beliefs about your medical care or treatment? For example, what kind of tests, procedures, or medications you prefer	3,493 (2.5%)	57,164 (40.9%)	79,088 (56.6%)
How often were you treated with respect by your doctors or health care providers?	1,296 (0.9%)	4,778 (3.4%)	133,671 (95.7%)
How often did your doctors or health care providers tell or give you information about your health and health care that was easy to understand?	1,664 (1.2%)	8,534 (6.1%)	129,547 (92.7%)

**Table 2:** Cohort demographics for each predictor variable. Includes all participants that answered at least one question in the Healthcare Access and Utilization survey

	*N*	Percent
Total Participants	165,812	100%
Genetic Condition		
Yes	7,106	4.3%
No	158,706	95.7%
Race		
White	134,248	81.0%
Black or African American	18,442	11.1%
Asian	6,482	3.9%
More than one population	3,747	2.3%
Another single population	1,138	0.7%
None of these	1,755	1.1%
Gender Identity		
Female	105,562	63.7%
Male	57,250	34.5%
Other Identity or Prefer Not to Answer	3,000	1.8%
Education Level		
High School Graduate or Below	19,295	11.6%
Some College through Advanced Degree	144,998	87.4%
Skip or Prefer Not to Answer	1,519	0.9%
Age at Analysis		
18–44	47,570	28.7%
45–64	55,576	33.5%
65+	62,666	37.8%
Annual Income		
Less than 10k to 35k	34,451	20.8%
35k to 100k	58,410	35.2%
100k to Over 200k	56,607	34.1%
Skip or Prefer Not to Answer	16,344	9.9%
Health Insurance Coverage		
Yes	159,875	96.4%
No	4,190	2.5%
Skip, Don’t Know, or Prefer Not to Answer	1,733	1.0%
Delayed Care due to Living in a Rural Area		
Yes	5,116	3.1%
No	154,567	93.2%
Skip or Don’t Know	6,129	3.7%

Counts and percentages by complete data for each survey question are available in Supplementary Tables 4–6. While primary inference was based on binary encoding of outcome variables, response counts across the four possible responses are also presented in Supplementary Tables 4–6.

**Fig. 2:**
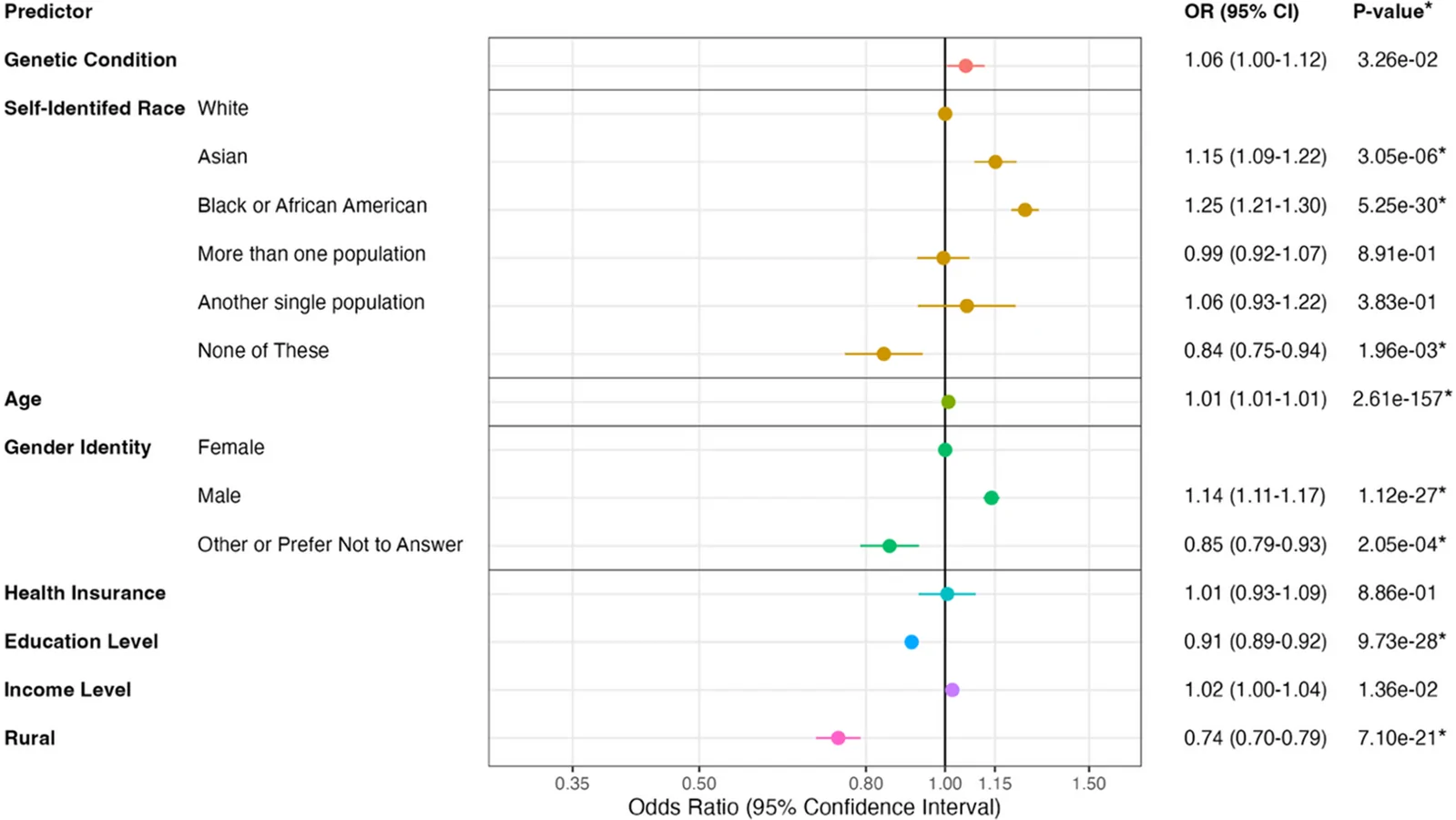
Identities and Factors Associated with Involvement in Health Care Decisions. Odds ratios with 95% confidence intervals are shown for the multivariable model. Note, the same X-axis scale is used for each survey question’s forest plot. P-values meeting significance of *p* < 0.002 denoted with an asterisk (*). The largest positive effect for this model was observed for participants identifying as Black or African American with approximately 25% increase in the odds of reporting involvement in health care decisions (OR = 1.25), compared to individuals identifying as white. Conversely, individuals who delayed care due to living in a rural area showed the largest negative effect with an approximate 26% decrease in the odds of reporting involvement in health care decisions (OR = 0.74), compared to individuals who did not report having to delay care

The first area of healthcare assessed was how patients would rate their involvement in decisions regarding their health care, through the following survey question: “How often did your doctors or health care providers ask for your opinions or beliefs about your medical care or treatment? For example, what kind of tests, procedures, or medications you prefer.” Of the three questions analyzed within this study, this question was the most divided with 42% (*n* = 57,164) of participants indicating low involvement in decisions regarding their healthcare (Table 1, Supplementary Table 4). Several variables (Fig. 2; Supplementary Table 7) met statistical significance for exhibiting a positive association with inclusion in healthcare decisions: identifying as Asian (OR = 1.15(1.09–1.22); *p* = 3.05 × 10^− 06^) or African American (OR = 1.25 (1.21–1.30); *p* = 5.25 × 10^− 30^), being older (OR = 1.01(1.01–1.01); *p* = 2.61 × 10^− 157^), or identifying as male (OR = 1.14(1.11–1.17); *p* = 1.12 × 10^− 27^). In contrast, variables that were associated with being involved less frequently included identifying as a race not listed in the survey options (OR = 0.84(0.75–0.94); *p* = 0.002), a gender identity other than female or male (OR = 0.85(0.79–0.93); *p* = 2.05 × 10^− 04^), higher education level (OR = 0.91(0.89–0.92); *p* = 9.73 × 10^− 28^), or having delayed care due to living in a rural area (OR = 0.74(0.70–0.79), *p* = 7.10 × 10^− 21^). This survey question was also analyzed as an ordinal outcome (four response levels); however, the overall inference and influence of predictors did not change from the logistic model, showing largest effects for contrasts of the top and lowest levels of response (Supplementary Table 8).

**Fig. 3:**
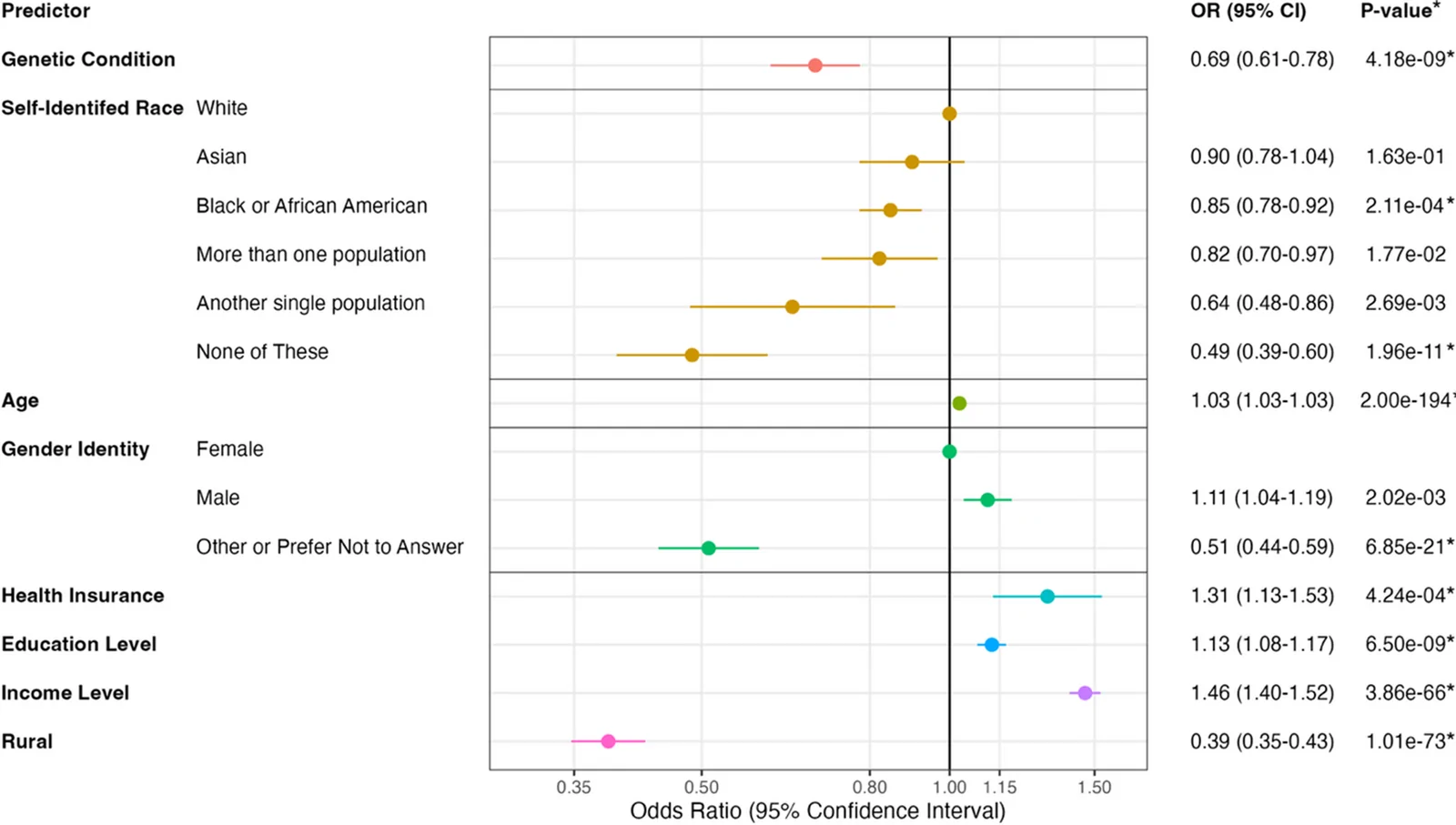
Identities and Factors Associated with Being Treated with Respect by Healthcare Providers. Odds ratios with 95% confidence intervals are shown for the multivariable model. Note, the same X-axis scale is used for each survey question’s forest plot. *P*-values meeting significance of *p* < 0.002 denoted with an asterisk (*). Across the three outcomes, this survey question showed the strongest statistical association for participants with a genetic condition, with an approximate 31% decrease in the odds of reporting being respected by a healthcare provider (OR = 0.69; *p* = 4.18 × 10^− 9^) compared to not having a genetic condition. The odds of reporting being respected by healthcare providers increased the most with income, with an approximate 46% increase in the odds for each increase of income level (OR = 1.46; *p* = 1.01 × 10^− 73^)

The second survey question assessed the frequency that participants felt respected by their healthcare providers: “How often were you treated with respect by your doctors or health care providers?” This question yielded a largely positive response with only 3.5% (*n* = 4,778) indicating negative experiences (Table 1, Supplemental Table 5). Predictor variables associated with feeling more respected by providers included being older (OR = 1.03(1.03–1.03); *p* = 2.00 × 10^− 194^), identifying as male (OR = 1.11(1.04–1.19); *p* = 0.002), having health insurance (OR = 1.31(1.13–1.53) *p* = 4.24 × 10^− 4^), higher educational attainment (OR = 1.13(1.08–1.17); *p* = 6.50 × 10^− 9^), and a higher annual income (OR = 1.46(1.40–1.52); *p* = 3.86 × 10^− 66^) (Fig. 3; Supplementary Table 9). There were several variables associated with feeling less respected by healthcare providers, including having a genetic condition (OR = 0.69(0.61–0.78); *p* = 4.18 × 10^− 9^), a gender identity other than female or male (OR = 0.51(0.44–0.59); *p* = 6.85 × 10^− 21^), or having delayed care due to living in a rural area (OR = 0.39(0.35–0.43); *p* = 1.01 × 10^− 73^). Compared to individuals self-identifying as White, many groups reported feeling less respected: identifying as another single population (OR = 0.64(0.48–0.86); *p* = 0.002), Black or African American (OR = 0.85(0.78–0.92); *p* = 2.11 × 10^− 4^, or a race not listed (OR = 0.49(0.39–0.60); *p* = 1.96 × 10^− 11^).

**Fig. 4:**
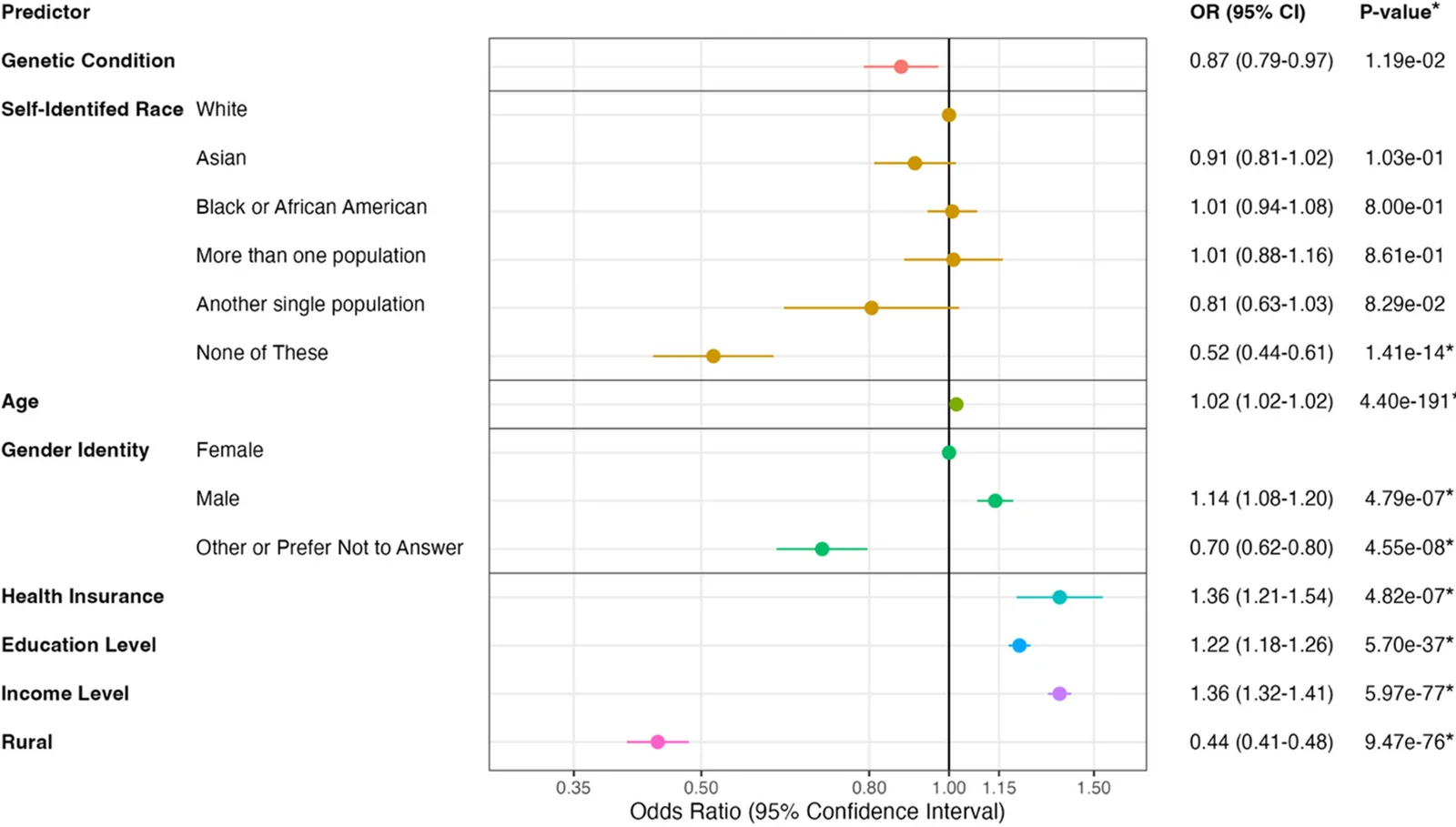
Identities and Factors Associated with Receiving Healthcare Information that was Easy to Understand. Odds ratios with 95% confidence intervals are shown for the multivariable model. Note the same X-axis scale is used for each survey question’s forest plot. *P*-values meeting significance of *p* < 0.002 denoted with an asterisk (*). As with each of the survey questions, having delayed care due to living in rural location again exhibited the greatest decrease in the odds of reporting positive healthcare experiences, with an approximate 56% decrease in the odds of receiving easy to understand healthcare information (OR = 0.44; *p* = 9.47 × 10^− 76^) compared to those who did not report delaying care due to living in a rural area

The last aspect of the healthcare experience we assessed was the frequency that individuals reported receiving information pertaining to their health that was easy to understand, through the following survey question: “How often did your doctors or health care providers tell or give you information about your health and health care that was easy to understand?” In total, 6.2% (*n* = 8,534) of participants indicated that they received information that was *not* easy to understand (Table 1, Supplemental Table 6). The following variables were positively associated with receiving information that was easy to understand: being older (OR = 1.02(1.02–1.02); *p* = 4.40 × 10^− 191^), identifying as a male (OR = 1.14(1.08–1.20); *p* = 4.79 × 10^− 7^), having health insurance (OR = 1.36(1.21–1.54); *p* = 4.82 × 10^− 7^), higher educational attainment (OR = 1.22(1.18–1.26); *p* = 5.70 × 10^− 37^), and a higher annual income (OR = 1.36(1.32–1.41); *p* = 5.97 × 10^− 77^) (Fig. 4; Supplementary Table 10). In contrast, individuals identifying as a race not listed (OR = 0.52(0.44–0.61); *p* = 1.41 × 10^− 14^), having a gender identity other than male or female (OR = 0.70(0.62–0.80); *p* = 4.55 × 10^− 8^), or having delayed care due to living in a rural area (OR = 0.44(0.41–0.48); *p* = 9.47 × 10^− 76^) were all associated with less frequently receiving understandable healthcare information. While having a genetic condition did not meet statistical significance when adjusting for multiple comparisons, the effect also trended towards less frequently receiving healthcare information that is easy to understand (OR = 0.87(0.79–0.97); *p* = 0.012).

Across the three models, “Patients Reported being Involved in Health Care Decisions” had the lowest predictive performance (c statistic = 0.56) compared to models for ‘Respect by Healthcare Providers’ (c = 0.71) and ‘Understanding of Healthcare Information’ (c = 0.67). For each of the three models, the magnitudes of effect for predictors were mostly reduced compared to the simple (single-variable) logistic regression models indicating that many of these variables are positively confounded with one another (Supplementary Tables 7, 9, 10). A main exception was for Survey question 2 (feeling respected by healthcare providers) for individuals having a genetic condition or reporting “none of these” for self-reported race indicating that these effects are potentially independent of the other predictors accounted for within this model.

Pairwise concordance between survey questions **(**Supplementary Table 11) were computed to assess consistency of positive or negative experiences across aspects of healthcare. Lowest correlations were identified between questions focused on patient involvement in healthcare decisions and feeling respected by healthcare providers (Cramér’s V = 0.165) as well as patient involvement in healthcare decisions and receiving healthcare information that was easy to understand (Cramér’s V = 0.238). There was a moderate correlation between patients feeling respected and receiving easy to understand healthcare information, with 93.5% of participants answering concordantly (Cramér’s V = 0.300) and most (92%) indicating positive responses for both questions.

## Discussion

While the three survey questions yielded largely positive experiences in aggregate, our analyses highlight several inequities which appear persistent in healthcare experiences across specific participant sociodemographic characteristics. Having health insurance, more educational attainment, and higher annual income were associated with reporting more positive experiences in terms of being treated with respect by providers and receiving easy to understand information. This finding mirrors prior literature suggesting individuals with more resources (e.g., income) report more positive health care experiences (Okunrintemi et al. 2019). However, we found that health insurance and income were not associated with patient involvement in health care decisions, and having a higher education level was associated with less decision involvement. It is possible that individuals with higher education levels have higher baseline trust of medical professionals and therefore feel less need for involvement, or that they more readily recognize when their provider did not present all options or involve them in all decisions. It is also possible that individuals with health insurance have variable experiences largely based on their provider’s perception or experiences with their insurance coverage, including that their “decisions” can be limited by what their insurance will cover. The greatest variability of responses was observed for “Involvement in Health Care Decisions” as 40% of participants reported being involved in their healthcare decisions only some of the time or less. While patient’s expectations regarding being involved in healthcare decisions can vary widely based on personal and cultural values and beliefs this statistic highlights an important reminder for health care providers and systems to ensure care is being provided in an individualized, patient-centric manner (Hawley and Morris 2017). One unexpected finding in our study, compared to prior literature, is that participants identifying as Black or African American reported the most frequent involvement in their healthcare decisions by their providers, which may have supported their decision to participate. Particularly given the distinctions between the demographics of the overall All of Us database compared to this study, it is likely that the self-selection of participants may have led to more positive survey responses compared to individuals who did not choose to complete the additional survey (Nguyen et al. 2020; Shepherd et al. 2018). It is critical to consider the impact of self-selection on this study, and that there is a large population of individuals who chose not to participate or may have had no awareness of the study, potentially related to limited accessibility, medical distrust, or countless other considerations. Over 90% of participants reported positive experiences in terms of being treated with respect by providers and receiving easy to understand information. This high percentage is encouraging, yet our analysis showed that individuals from underrepresented backgrounds continue to report more negative experiences in both areas, therefore highlighting a valuable area for community-based research to ensure all patients feel they are being treated with respect and are receiving understandable healthcare information.

Additionally, being older and identifying as male were associated with more positive healthcare experiences across all three survey questions. In stark contrast, identifying as a race group that was not listed, having a gender identity other than female or male binary, and having delayed care due to living in a rural location were associated with poorer healthcare experiences in all three areas explored. Furthermore, almost every group of participants that did not identify as White reported feeling respected significantly less frequently by providers. These prior negative healthcare experiences may represent key factors contributing to underrepresentation in research among rural residents and racial, ethnic, and gender minorities due to the fostering of medical distrust, as individuals may anticipate being treated poorly if they enroll in medical research such as genetic studies. However, future research is needed to test this hypothesis. These findings also point towards the need for culturally competent and patient-oriented healthcare training programs, continued learning opportunities, and support resources, ideally developed in partnership with underrepresented groups, for health care professionals to learn how to best support and meet diverse patient needs (Adsul et al. 2025; Michener et al. 2025). As previously noted, desired involvement in healthcare decisions may be very individualized, so providers may want to establish their patients’ desired level of inclusion in medical decisions.

Interestingly, individuals diagnosed with genetic conditions reported varying healthcare experiences across the three survey items. Having a genetic condition positively trended with inclusion in healthcare decisions but negatively trended with receiving easy to understand information. Notably, the largest (and statistically significant) negative effect was identified for question 2, where patients with a genetic condition exhibited an approximate 31% decrease in the odds of reporting being respected by healthcare providers. The concordance analysis, across all participants, provided additional evidence that receiving healthcare information in an easy-to-understand way was correlated with patients feeling being respected. The complexity of genetics, particularly regarding genetic testing and result interpretation, could contribute towards patients with genetic conditions reporting that they receive information about their healthcare that is difficult to understand. This result highlights that there is continued room for improvement and research within the field of medical genetics to understand how to best support patients in understanding such complex information about their health. As access to genetic healthcare increases, the number of individuals diagnosed with genetic conditions is also expected to increase; thus, education about genetics and testing is critical and will need to adapt to the diverse backgrounds and prior knowledge of each patient. The overall findings indicate that many individuals who are part of historically underrepresented groups continue to have disparate healthcare experiences in each of the areas assessed, correlating with the continued lack of representation in current research studies. These findings support the proposed conceptual model of the cycle of genomic healthcare disparities and reinforce the need for continued work is necessary to improve not only access, trust, and equitable representation in research, but also healthcare accessibility and experiences.

### Limitations and considerations

Our study has important limitations. Overall, there were reduced percentages of underrepresented groups among those that had taken the “Healthcare Access and Utilization” survey as compared to the total *All of Us* dataset. Within the HCAU cohort, we found increased numbers of individuals reporting to be white, having higher educational attainment, and/or having higher incomes. This presents biases, relative to the general population, likely inflating the high percentages of positive experiences in response to survey questions. Interestingly, having a genetic condition was not strongly biased in our cohort, relative to the full All of Us v7 data. Data analysis was also limited by what information was made available within the released dataset, notably assessment of the healthcare experiences of individuals who did not identify as male or female was impacted due to being grouped with those who elected not to answer. It is also important to note that these findings may not be reflective of the experiences of individuals who have had poor enough experiences to avoid accessing healthcare or who distrust their providers and/or the research process. Additionally, over 90% of participants reported feeling respected by healthcare providers and that they were given understandable healthcare information, which may have played a role in their decision to participate in the *All of Us* research study. Further, identification of individuals in the “diagnosed genetic condition” group relied on both the participant sharing electronic medical record data as well as the provider having documented this diagnosis in their chart. Therefore, it is plausible that there were additional individuals in the study who had a genetic diagnosis or who are currently undiagnosed due to the barriers in accessing genetic healthcare. Despite these limitations, our study is strengthened by the large sample size, as we were statistically powered to detect even small effect sizes within this study of over 135,000 individuals. Given the large sample size, we were able to better demonstrate significant effects even within smaller populations, including being well-powered to assess variables associated with negative experiences. We also note that due to the statistical power of the study some highly significant associations may not be as clinically impactful due to small effects sizes (e.g., age with OR = 1.01; *p* = 2.61 × 10^− 157^ for survey question 1). For this reason, we focused on variables with larger effects sizes.

### Future directions

Based on our findings, there are several future studies that could better define not only the proposed conceptual model of the genomic healthcare disparity cycle but also begin the process of developing resources to break down the cycle as we strive for equitable and personalized healthcare. As many individuals participating in the *All of Us* study submit samples for clinical genetic testing, further analysis of these results to identify individuals with previously undiagnosed genetic conditions and assessment of the groups most impacted could indicate whether poorer healthcare experiences could be intertwined with lack of diagnoses. Analyzing the impact of the social determinants of health and their role in impacting healthcare experiences between individuals with and without genetic diagnoses could further inform our discussion about the additional barriers that individuals face in attaining genetic healthcare. Specifically, there are additional barriers covered by the HCAU survey that could be explored, including more granular consideration of healthcare coverage, beyond a simple dichotomous outcome. This would facilitate exploration of the other dimensions within the Genomic Healthcare Disparity Cycle: Healthcare Accessibility and “Inter/Intrapersonal Values and Beliefs”. Community-based studies with family groups dedicated to one or a group of genetic conditions could help identify patients and families who may benefit most from increased resources and provider education to improve their healthcare experiences (Lemke et al. 2022; Michener et al. 2025). Finally, for the groups identified in this study reporting more negative healthcare experiences, qualitative studies exploring what they perceive would improve these experiences could aid in the development of clinical and patient resources that would be most beneficial for them.

## Conclusion

In conclusion, our findings suggest that many groups who continue to be underrepresented in genomic research also face inequities in their healthcare experiences. Future work is needed to understand and solidify the role these experiences play in the genomic healthcare inequity cycle and to improve healthcare experiences for all patients. Further, the groups that experience the greatest healthcare experience disparities in this study included individuals who did not identify with the listed race options, individuals with a gender identity other than male or female, and particularly people in rural areas. To provide equitable healthcare, genomic medicine and the wider community of healthcare providers should strive to address these disparities in healthcare experiences. The importance of striving for continued improvement of community access to genetic healthcare cannot be understated, and to address the root causes of these disparities it is critical to seek the insight of individuals who are part of these groups in developing resources that will be truly beneficial. As the cycle of genomic healthcare disparities suggests, individuals with inequitable access to and experiences while receiving healthcare likely experience more hesitation surrounding participation in research. Since this continued limited research participation can unfortunately contribute towards continued inequities within genetics, this study emphasizes the importance of focusing on the larger healthcare experience due to the potential role it plays in both research participation and overall health outcomes.

## Supplementary Information

Below is the link to the electronic supplementary material.


Supplementary Material 1


## Data Availability

The data that support the findings of this study are available from All of Us Research Hub. Restrictions apply to the availability of these data, which were used under license for this study. Data is available at https://www.researchallofus.org/ with the permission of All of Us.
